# Multimarker Screening of Oxidative Stress in Aging

**DOI:** 10.1155/2014/562860

**Published:** 2014-07-16

**Authors:** Kamila Syslová, Adéla Böhmová, Miloš Mikoška, Marek Kuzma, Daniela Pelclová, Petr Kačer

**Affiliations:** ^1^Institute of Chemical Technology, Technicka 5, 166 28 Prague 6, Czech Republic; ^2^Department of Occupational Medicine, First Faculty of Medicine, Charles University in Prague and General University Hospital in Prague, Na Bojisti 1, 120 00 Prague 2, Czech Republic

## Abstract

Aging is a complex process of organism decline in physiological functions. There is no clear theory explaining this phenomenon, but the most accepted one is the oxidative stress theory of aging. Biomarkers of oxidative stress, substances, which are formed during oxidative damage of phospholipids, proteins, and nucleic acids, are present in body fluids of diseased people as well as the healthy ones (in a physiological concentration). 8-*iso* prostaglandin F_2*α*_ is the most prominent biomarker of phospholipid oxidative damage, *o*-tyrosine, 3-chlorotyrosine, and 3-nitrotyrosine are biomarkers of protein oxidative damage, and 8-hydroxy-2^′^-deoxyguanosine and 8-hydroxyguanosine are biomarkers of oxidative damage of nucleic acids. It is thought that the concentration of biomarkers increases as the age of people increases. However, the concentration of biomarkers in body fluids is very low and, therefore, it is necessary to use a sensitive analytical method. A combination of HPLC and MS was chosen to determine biomarker concentration in three groups of healthy people of a different age (twenty, forty, and sixty years) in order to find a difference among the groups.

## 1. Introduction

Aging is a multifactorial process of time-dependent decline in physiological function [1]. It is manifested by the decrease of the efficiency of the organism functions, the accumulation of various defects and declining ability to repair them, increased susceptibility to various diseases, and eventually increased mortality [2, 3].

Many theories explain the phenomenon of aging. The most popular one is the free radical theory which was proposed by Harman in 1956 [4]. Harman suggested that OH and OH_2_ radicals are produced endogenously in living organisms during oxygen-utilizing processes (such as respiration). Later on, it was found that there are other oxygen compounds such as hydrogen peroxide or hypochlorous acid which react with biomolecules in the same way. These are, together with oxygen radicals, called reactive oxygen species (ROS). Considering this, the free radical theory was modified to oxidative stress theory of aging [5, 6].

Oxidative stress is defined as an imbalance between oxidants (ROS) and the antioxidant defense in the organism in favor of oxidants [7]. The oxidants interact with biomolecules in cells such as phospholipids, proteins, and nucleic acids. This leads to cell dysfunctions and consequently cell death. The molecules formed during oxidation may serve as biomarkers as their analysis in various biological matrices is used for the quantification of oxidative stress in humans. The most significant biomarker of oxidative stress is 8-*iso* prostaglandin F_2*α*_ (8-isoprostane). 8-Isoprostane is formed by nonenzymatic oxidation of arachidonic acid. Oxidation of proteins and amino acids gives rise to* o*-tyrosine, 3-chlorotyrosine, and 3-nitrotyrosine. FENO (fractional exhaled nitric oxide) present in EBC is also formed from amino acid, L-arginine, by its oxidation; elevated or even depressed level of FENO is linked with asthma, upper airway infections, and other lung diseases [8, 9]. Biomarkers of nucleic acid oxidation are 8-hydroxyguanosine and 8-hydroxy-2′-deoxyguanosine. High concentrations of the biomarkers were determined not only in body fluids or tissues of patients with age-related and/or degenerative diseases such as Alzheimer's disease, hypertension, type II diabetes, or several types of cancer (see Table 1 for the summary of diseases and detected biomarkers) but also in relation to chronic obstructive pulmonary disease [10], smoking [11, 12], and air pollution [13].

## 2. Biomarkers of Oxidative Stress

### 2.1. 8-Isoprostane

**Figure 1:**
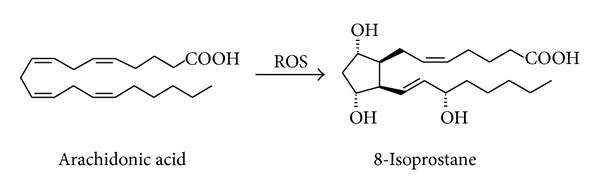
The formation of 8-isoprostane from arachidonic acid.

8-Isoprostane is formed by nonenzymatic oxidation of arachidonic acid (Figure 1) which is present in phospholipid membranes [40]. A similar metabolic pathway, the enzymatic *ω*-hydroxylation of arachidonic acid in the presence of an increased cytochrome P450 4A, owing to organism aging, leads to a similar compound, that is, 20-hydroxyeicosatetraenoic acid (20-HETE), as a very potent vasoconstriction agent [41].

Although it was thought that 8-isoprostane acts only through thromboxane (TP) receptors, the biological activity of 8-isoprostane is slightly different which suggests the existence of a specific isoprostane receptor. Incubation of 8-isoprostane with platelets causes only shape changes of platelets and in very high concentrations a reversible aggregation, while thromboxane A_2_ (TXA_2_) causes an irreversible aggregation of platelets [43]. Isoprostanes have a strong vasoconstriction effect also partly by influencing TP receptors but have stronger influence on renal vasoconstriction and weaker influence on bovine coronary arteries than TXA_2_ agonists [44, 45]. According to these findings, a hypothesis for existence of specific isoprostane receptor on smooth muscle cells in vascular system has been proposed. Other studies showed that there are high-affinity and low-affinity binding sites for 8-isoprostane on smooth muscle cells in vascular system and on endothelium cells. Low-affinity binding sites could represent TP receptors and high-affinity binding sites specific isoprostane receptors [46]. In conclusion, 8-isoprostane causes vasoconstriction of blood vessels and bronchi, lowers blood flow in kidneys, influences aggregation of platelets, and, thus, participates in pathology of several diseases (Table 2).

Concentration of 8-isoprostane in body fluids is used for the monitoring of oxidative stress. Higher concentration levels were observed, for example, in smokers (24 ± 8 pg/mL) and patients with cystic fibrosis (43 ± 7 pg/mL) compared to healthy nonsmokers (11 ± 4 pg/mL) [47] and also in arthritis [20], age-related cataracts [28], hypertension [31, 32], asthma [48, 49], and type II diabetes [36, 37].

### 2.2. *o*-Tyrosine and* m*-Tyrosine

In the organism, tyrosine is formed from phenylalanine. Physiological* p*-tyrosine occurs by enzymatic oxidation of phenylalanine by phenylalanine hydroxylase. Important derivates of tyrosine are catecholamines (dopamine, adrenaline, and noradrenaline) or thyroid hormones.

**Figure 2:**
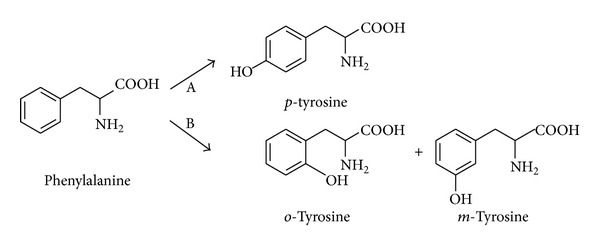
The formation of tyrosine from phenylalanine. A: enzymatic oxidation; B: oxidation by hydroxyl radicals.

*o*-Tyrosine (*o*-Tyr) and* m*-tyrosine (*m*-Tyr) are formed by the attack of ROS on phenylalanine (Figure 2). Unlike* p*-tyrosine,* o*-Tyr and* m*-Tyr are not natural amino acids and are considered to be oxidative stress biomarkers.

Estrogen receptor *α* (ER*α*) is a nuclear protein which is overexpressed in breast cancer cells [50]. The anticancer property of chlorambucil linked to estradiol was observed [51]. Nevertheless, estrogenic drug can not only target the cancer cells but also induce transcriptional activity [52]. Such estrogenic activity could be avoided by choosing nonsteroidal drugs with structural similarities. Tyrosine shows some structural similarities with estradiol and the phenol group was found important for binding to ER*α*. The affinity of tyrosine-chlorambucil derivate to ER*α* was tested in order to investigate the role of the position of the hydroxyl group (use of* o*-tyrosine,* m*-tyrosine, and* p*-tyrosine). All compounds with tyrosine showed higher cytotoxicity than pure chlorambucil. Compared to the other regioisomers, the* m*-tyrosine-chlorambucil compound showed greater cytotoxicity and it was also slightly more specific for hormone-dependent cancer cells, probably due to closer similarity to estradiol [53].

Higher concentration of* o*-Tyr was found, for example, in lenses of patients with cataracts [29] and in blood plasma and urine of patients with type II diabetes [39]. Elevated levels of* m*-Tyr were confirmed in lenses of patients with cataracts [29].

### 2.3. 3-Chlorotyrosine and 3-Nitrotyrosine

**Figure 3:**
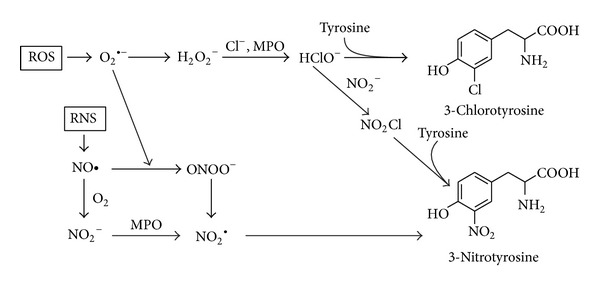
The formation of 3-chlorotyrosine and 3-nitrotyrosine by myeloperoxidase (MPO), adapted from [42].

3-Chlorotyrosine (3-ClTyr) is formed by the reaction of hypochlorous acid (HClO) and* p*-tyrosine (Figure 3). Hypochlorous acid is formed from hydrogen peroxide and chloride anion by myeloperoxidase (MPO) as a catalyst. MPO is a phagocyte heme protein, catalyses the transformation of hydrogen peroxide (H_2_O_2_) and chloride anion (Cl^−^) into highly reactive hypochlorous acid, and plays an important role in the microbicidal activity of phagocytes [54, 55].

MPO causes also nitration of tyrosine (the formation of 3-nitrotyrosine). 3-Nitrotyrosine (=3 NOTyr) can also be formed by the reaction of peroxynitrite (ONOO^−^) and* p*-tyrosine in proteins (Figure 3).

The formation of 3-ClTyr in proteins plays an important role in cardiovascular system. HDL (high-density lipoprotein) and its major protein, apolipoprotein A-I (apoA-I), are thought to protect the organism against atherosclerosis. One of the mechanisms is the removal of excess intracellular cholesterol from macrophages [56]. The removal is controlled by ATP-binding cassette transporter (ABCA1) [57], a membrane protein that exports cholesterol from cells to apoA-I. ABCA1 is induced by intracellular cholesterol and is highly expressed in cholesterol-loaded cells (such as foam cells in early atherosclerosis lesions) [58]. The cholesterol removal requires (1) direct binding of apoA-I to ABCA1 [59, 60], (2) solution of lipid domains formed by ABCA1 in cell membrane by apoA-I [61–64], and (3) activation of several signaling pathways. It was shown that MPO-mediated chlorination of apoA-I impairs the direct binding of apoA-I to ABCA1 and, thus, contributes to atherogenesis by impairing cholesterol efflux from macrophages [65].

It was also found that free 3-ClTyr promotes the migration of human aortic smooth muscle cells (the major mechanism of the vascular lesion formation) and that increased levels of 3-ClTyr under inflammation conditions may contribute to vascular diseases [66].

Also nitration of proteins changes the function of proteins. Nitration of tyrosine lowers the pK_*a*_ from 10.0–10.3 to 7.2–7.5 [67] and, thus, changes the pI of a protein; 3-NOTyr containing proteins are more hydrophobic [68]; and the nitrogroup is a relatively bulky substituent, which may add steric restrictions to the molecule of protein [69]. However, only a limited number of proteins constitute preferential target to nitration and only few tyrosines can be nitrated within a protein [70], but several common features of tyrosine nitration have been revealed: (1) the presence of one or more acidic residues in the vicinity of the target tyrosine (glutamic or aspartic residues), (2) the small number of cysteine or methionine residues adjacent to the nitrated tyrosine residue, and (3) the presence of turn-inducing amino acids such as proline and glycine [70, 71]. In the organism, posttranslational modification such as nitration can cause (1) no change in protein function, (2) loss of function, or (3) gain of function. The loss of function was demonstrated, for example, on MnSOD (manganese superoxide dismutase, a mitochondrial enzyme) [72] or PGI_2_ (prostacyclin (prostaglandin I_2_) synthase, a vascular enzyme) [73]. The gain of function was demonstrated, for example, on cytochrome* c*, which gains peroxidase activity [74, 75]; on fibrinogen (higher aggregation in coagulation) [76]; or on protein kinase C*ε* [77] (summary in Table 3, adapted from [71]).

Higher concentration of 3-ClTyr was found in patients with Alzheimer's disease. These patients have higher activity of MPO, increased formation of hypochlorous acid, and therefore higher concentration of 3-ClTyr [14]. In the blood plasma, 3-ClTyr can bind to HDL and LDL (high- and low-density lipoprotein) and thus cause the progress of atherosclerosis. The concentration of 3-ClTyr in LDL of patients with atherosclerosis was 30 times higher compared to healthy people [25]. 3-ClTyr serves also as a biomarker of MPO increased activity because it is not formed by other mechanisms and is stable at elevated temperature [42].

Higher concentrations of 3-NOTyr were found in cerebrospinal fluid [20, 21] of Alzheimer's disease patients. The concentration of 3-NOTyr was 11.4 ± 5.4 nM in patients and 1.6 ± 0.4 nM in the group of healthy volunteers [16]. Besides, elevated 3-NOTyr levels can be found in patients with arthritis [21, 22], atherosclerosis [26], and hypertension [33].

### 2.4. Advanced Oxidation Protein Products

Extracellular fluids contain only minor amounts of antioxidant enzymes and thus plasma proteins (e.g., albumin) are prone to oxidation by ROS. Elevated levels of oxidized protein products are termed “advanced oxidation protein products” (AOPP). AOPP are produced by the myeloperoxidase- (MPO)-H_2_O_2_-halide system of activated phagocytes. First step of this reaction is oxidation of coenzyme NADPH by hydrogen peroxide. During this reaction hypochlorous acid (HOCl) is produced. The Cl^−^ ion is used as a substrate by the MPO enzyme. Myeloperoxidase is produced from hydrogen peroxide activated leukocytes. The generation of cytotoxic HOCl also causes the formation of advanced oxidation protein products (AOPP) by attacking normal tissue with consequent protein oxidation.

Higher concentrations of AOPP were found in plasma or urine of patients with acute coronary syndrome or active ulcerative colitis. The concentration of AOPP in plasma was determined by 140–180 *μ*M for patients and 60–70 *μ*M for the group of healthy volunteers [83, 84].

### 2.5. 8-Hydroxy-2′-deoxyguanosine and 8-Hydroxyguanosine

**Figure 4:**
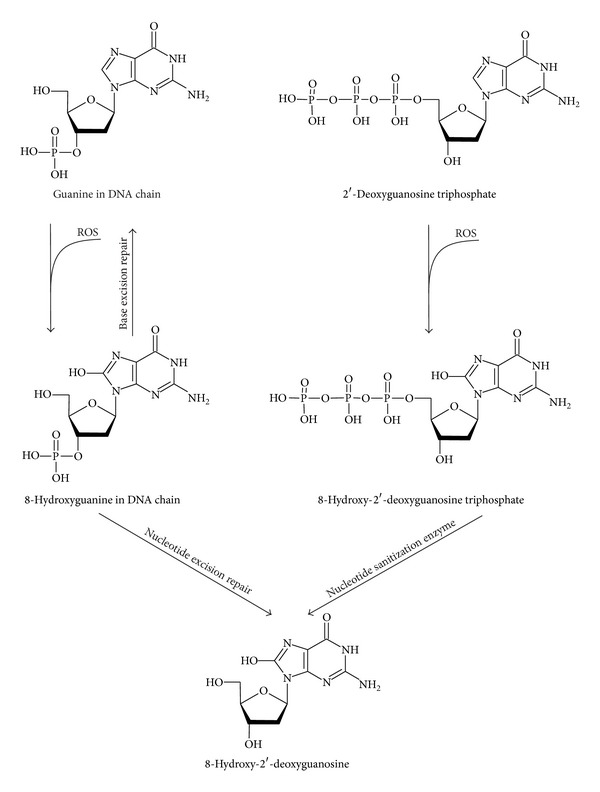
The formation of 8-hydroxy-2′-deoxyguanosine.

**Figure 5:**
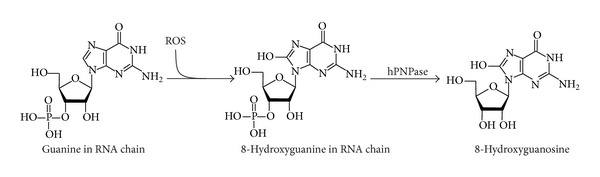
The formation of 8-hydroxydeoxyguanosine.

8-Hydroxy-2′-deoxyguanosine (8-OHdG; Figure 4) is the main product of DNA oxidation. 8-Hydroxyguanosine (8-OHG; Figure 5) is formed by oxidation of RNA.

Two mechanisms are possible for the release of 8-OHdG to urine and blood plasma. First, 2′-deoxyguanosine triphosphate and hydroxyl radical form 8-hydroxy-2′-deoxyguanosine triphosphate which is enzymatically transformed to 8-hydroxy-2′-deoxyguanosine monophosphate (8-OHdGMP). 8-OHdG is released by digestion of 8-OHdGMP. Second, 8-OHdG is formed by nucleotide excision repair (NER) mechanism. The whole sequence containing damaged nucleic base (oligonucleotide) is removed from DNA and the missing part of the strand is synthesized according to the other complementary strand [85].

8-OHG is formed by the reaction of RNA and hydroxyl radical [86]. Human polynucleotide phosphorylase (ribonuclease hPNPase) is assumed to remove 8-OHG from RNA [87]. Oxidation of mRNA lowers the effectiveness of translation (synthesis of primary protein structure according to the genetic information in mRNA), induces formation of abnormal proteins [88], and is one of the primary factors causing cell death [89].

In rat model, 8-OHdG was found to have anti-inflammatory effect [90]. Rats treated with lipopolysaccharide (LPS) exhibited inflammatory lung injury dependent on neutrophils with increase in proinflammatory cytokines such as interleukins 6 and 18 (IL-6, IL-18) and tumor necrosis factor *α* (TNF-*α*). Rats pretreated with 8-OHdG prior to LPS treatment showed inhibited LPS-induced inflammatory responses. 8-OHdG anti-inflammatory action was found to be higher than that for aspirin and other nucleosides (8-OHG, deoxyguanosine, guanosine, and adenosine). 8-OHG and adenosine also exhibited anti-inflammatory activity, but it was much lower than that for 8-OHdG. Deoxyguanosine was found to be almost ineffective. Compared to aspirin, which acts through cyclooxygenase (COX) inhibition, 8-OHdG seems to be more versatile and, therefore, more effective as it was found that 8-OHdG suppresses ROS formation in human neutrophils. However, in humans, 8-OHdG is excreted in much lower concentrations than in rats and, therefore, only exogenously administered 8-OHdG could have a therapeutic potential as anti-inflammatory agent [90].

Higher concentration of  8-OHdG was found, for example, in patients with Alzheimer's disease [18], arthritis [23, 24], atherosclerosis [27], cataracts [30], hypertension [34], osteoporosis [35], or type II diabetes [38, 40]. 8-OHdG is also considered to be a potential biomarker of cancers related to smoking (e.g., lung cancer). The concentration was 1.57 ± 0.86 nM in patients with cancer and 1.09 ± 0.52 nM in the control group of healthy volunteers [91].

8-OHG can be found in patients with Alzheimer's disease and it has been shown that oxidative damage of RNA is higher than damage of DNA [18, 19]. The concentrations in cerebrospinal fluid for Alzheimer's disease were 500 ± 213 pM in the patients and 97 ± 32 pM in the control group. The difference of the concentration in blood serum was not significant [19].

## 3. Methods for Determination of Oxidative Stress Biomarkers

The complexity of biological matrices, different molecular structures of biomarkers, and variety of existing analytical methods gives us a lot of possibilities to determine the biomarkers. The most common analytical methods are summarized in Table 4.

Biochemical methods such as ELISA (enzyme-linked immunosorbent assay) and EIA (enzyme immunoassay) allow the determination of lower concentrations than methods combining chromatographic methods and mass spectrometry (GC-MS, HPLC-MS). However, the disadvantage of biochemical methods is the possibility of cross-reactions which cause false-positive or false-negative results [104]. Currently, the limit of detection (LOD) of methods combining chromatography and mass spectrometry is pico- and femtomoles per milliliters which are concentrations of biomarkers in body fluids. Additionally, both quantitative and qualitative (structure of the substance) information are gained. Therefore MS^n^ techniques, which have a high selectivity, are used more often.

ELISA (enzyme-linked immunosorbent assay), also called EIA (enzyme immunoassay), is one of the most frequently used methods applicable in the quantitative analysis of antigens. This method exists in a range of modifications which are all based on a highly specific interaction of antigen and antibody. One of these binding partners is covalently bound to an enzyme (usually peroxidase, acetylcholinesterase, or alkaline phosphatase) whose role is the catalytic conversion of the added substrate to a colored product. The color intensity, determined by spectrophotometric or fluorimetric methods, directly or indirectly reflects the amount of the antigen present in the sample. When the antigen is determined, the immobilization (via adsorption or a covalent bonding) of the antibody on a solid support is a common characteristic of all ELISA methods. The immobilization of antibodies (e.g., on a microtiter plate) enables the separation of antigens (biomarkers) from biological matrices (exhaled breath condensate, blood plasma, and urine).

Radioimmunoassay (RIA) works on a similar principle as ELISA. The main difference is in the use of a labeled antigen. The enzyme on the antigen is replaced by a tyrosine moiety containing a *γ*-radioactive iodine isotope. The *γ*-radiation is monitored by the presence of the nonbonded labeled antigen in the sample. The very sensitive and specific methods based on RIA for 8-isoprostane determination in EBC have been successfully developed and validated [104]. RIA is also very suitable for determination of FENO or prostaglandins in EBC from patients suffering from asthma or cystic fibrosis [105, 106]. However, radioactive species can be operated only in specialized laboratories with appropriate equipment, which is a relevant disadvantage and explains the less frequent utilization of RIA in practice.

For detection of proteomics markers of oxidative stress (AOPP), ELISA test [107, 108] or methods with mass spectrometric detection [104, 109, 110] can be used. During MS detection can be used protein digest method (digest of proteins to smaller peptides using a protease such as trypsin) or protein nondigest method (intact proteins are ionized by ESI or MALDI ionization and then introduced into a mass analyzer. This approach is referred to as “top-down” strategy of protein analysis).

Electronic nose as a novel analytical technique for determination of volatile compounds in EBC usually comprises an array containing a number of chemical sensors. The choice of the sensors represents difficult task due to their specificity, response and recovery time, range of compounds detected, sensitivity, operating temperature, physical size, temperature and humidity effect on sensor functioning, portability, and cost and circuitry complexity. The molecules of analyte are adsorbed on the sensor surface providing the signal that fades with desorption. The similarity to biochemical methods mentioned above and detection limits as low as tens of ppb make this innovative technique really promising [111, 112].

Nuclear magnetic resonance (NMR) is primarily intended for qualitative structural analysis although it has been proven to be a valuable tool for comparison of different groups of individuals and statistical evaluation of collected data using methods such as PCA (principle component analysis) for key biomarkers present in EBC [113, 114]. This method uses the interaction of strong magnetic field with atomic nuclei possessing nonzero spin. The signal is created via absorption of high frequency radiation causing specific spin energy distribution. NMR technique can be used for both proteomics and metabolomics [115, 116].

Gas chromatography coupled with mass spectrometry (GC-MS) can be used for the analysis of analytes giving information about both their structures and their quantities. This analytical method takes advantage of its (1) high separation selectivity determined by the type of capillary columns used and (2) high specificity and sensitivity enabled by the integration of the mass spectrometric detector. Therefore, the GC-MS method allows the quantification of substances in biological matrices or tissue on nanogram per milliliter or gram level. The most significant disadvantage is the need for a sufficient volatility and thermal stability of analytes in the sample. To resolve it, pretreatment procedures (extraction and derivatization) are necessary to be included in this particular case prior to quantitative and qualitative analysis. Derivatization is a chemical reaction of an analyte with a suitable derivatization reagent which changes its physical and chemical properties (in this case mainly volatility and thermal stability). Additionally, derivatization prior to a GC-MS analysis is carried out to improve the sensitivity of the MS detection by enabling a better fragmentation in the detector. For example, 3-NOTyr is measured as methyl ester-diheptafluorobutyl amide-methyl ether (Me-HFB-Me) derivative [117], di-*O*-methyldi-*N*-heptafluorobutyryl derivate [117],* n*-propyl-PFP-TMS derivative [118, 119], and pentafluorobenzyl derivate [120]. 3-ClTyr is measured as* N*(*O*)-ethoxycarbonyl trifluoroethyl amino acid ester [121].

**Figure 6:**
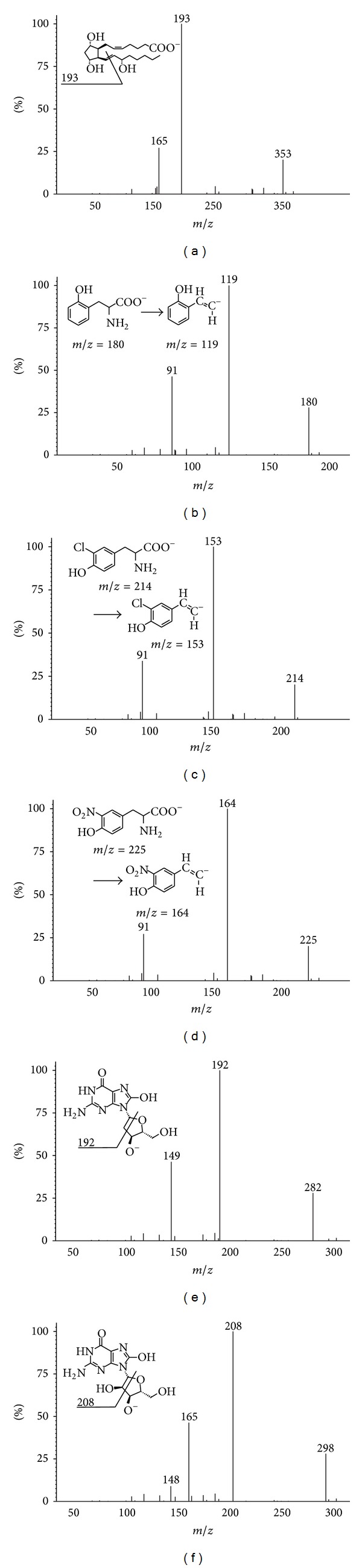
MS/MS spectra for (a) 8-isoprostane, (b)* o*-tyrosine, (c) 3-chlorotyrosine, (d) 3-nitrotyrosine, (e) 8-hydroxy-2′-deoxyguanosine, and (f) 8-hydroxyguanosine.

**Figure 7:**
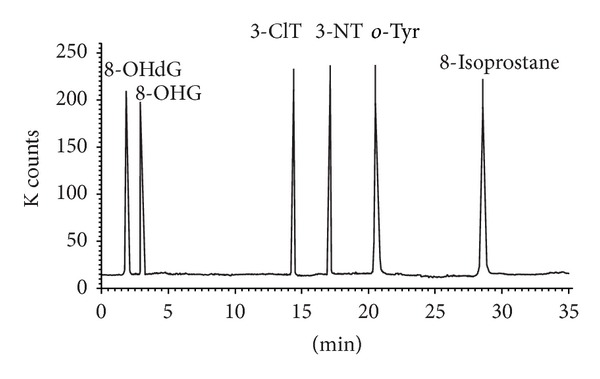
HPLC-ESI-MS/MS chromatogram.

High performance liquid chromatography (HPLC) in combination with mass spectrometry (MS) is generally used for the analysis of low volatile and thermally labile substances. The high selectivity of separation is achieved by a suitable choice of chromatographic phase systems, that is, the liquid and stationary phase. Reversed-phase HPLC is the most commonly used with the stationary phase consisting of silica gel modifiable by nonpolar octadecyl groups and the polar liquid phase usually consisting of water, acetonitrile, or methanol, optionally with addition of buffers. For the detection, usually UV, fluorescence, electrochemical, or MS methods are used. Nowadays, the combination of HPLC and MS allows facile separation and parallel detection of even very low analyte concentrations present in complex matrices. Since the remaining detectors mentioned above do not allow the quantification of analytes and lack the high specific structural information, HPLC-MS is becoming the first choice method for the analysis of substances in biological matrices. Therefore, the analysis of complex body fluids on a picogram scale is viable using HPLC-MS and also suitable for future routine practice. In order to increase the detector precision and sensitivity, the following is advisable prior to the HPLC-MS analysis: (1) the addition of an isotopically labeled internal standard and (2) the use of a pretreatment method (immunoextraction, solid phase extraction, and lyophilisation) to remove undesired species and concentrate the sample. When MS detection is utilized, the analytes need to be evaporated and ionized. As this can be carried out at atmospheric pressure (API: atmospheric pressure ionization), it is also feasible with thermally labile substances. Electrospray ionization (ESI) is one of the most frequently used API techniques. It is a soft ionization technique characterized by the preservation of a molecular ion peak with minimal fragmentation of the analyzed molecule. Depending on the molecule charge of a measured analyte, two measurement modes can be distinguished, that is, positive electrospray ionization (ESI^+^) in which protonated molecular ion [M+H]^+^ is produced and negative electrospray ionization (ESI^−^), where the molecule is deprotonated [M−H]^−^. The molecule ion ([M+H]^+^ or [M−H]^−^ ion (given chemical structure of the detected biomarkers)) is preferred for all determined biomarkers. The combination of ESI ionization and a triple-stage quadrupole analyzer (TSQ) is a suitable detection technique for the quantification of the analytes. The first and the third quadrupole (Q1 and Q3) are identical and capable of using the same scan modes. On the contrary, the second quadrupole (Q2) is different in both its construction and function, allowing the fragmentation of the analyte upon elastic collision with an inert gas (argon). Therefore, it is often referred to as the collision cell. A mass spectrometer equipped with a triple quadrupole uses a highly selective single reaction monitoring mode (SRM) for the quantification and structural identification of substances. In the case of oxidative stress biomarkers, Q1 isolates the deprotonated [M−H]^−^ molecular ions, which are further used as precursor ions for the subsequent collision-induced dissociation (CID) in Q2. In the collision cell, the molecule selectively degrades and yields product ions which are analyzed on quadrupole Q3 giving MS/MS spectra (Figure 6). Methods used for the quantification of the biomarkers are in Table 5. For HPLC, a gradient elution with flow rate of 200 *μ*L/min was used (Table 6) [97], mobile phase A was a water solution of ammonium hydroxide (pH = 10.5), and mobile phase B was a mixture of MeOH/ACN (60 : 40 v/v) with 0.1% ammonium hydroxide. The retention times were as follows: dead time of the column = 0.8 min; *R*_*t*_ (8-OHdG) = 1.9 min; *R*_*t*_ (8-OHG) = 3.1 min; *R*_*t*_ (3-ClTyr) = 14.4 min; *R*_*t*_ (3-NOTyr) = 17.0 min; *R*_*t*_ (*o*-Tyr) = 20.6 min; and *R*_*t*_ (8-iso) = 29.5 min (Figure 7).

## 4. Clinical Study

It is generally accepted that concentrations of oxidative stress biomarkers are increasing with increasing age. Although it has not been proven for every single known biomarker, several studies confirm initial statement [122]. The studies that have been published so far are generally not focused on relation between levels of biomarkers in healthy subjects and their age, but they are focused on monitoring of levels of oxidative stress biomarkers linked to particular disease (e.g., Alzheimer's disease and Parkinson's disease; see Table 1). Some authors [123, 124] have observed the elevation of specific oxidative stress biomarker in biological matrix, but so far there has not been performed wider metabolomic screening of oxidative stress biomarkers in relation to the age of healthy individuals.

We compared three groups of people with similar age. The first, labelled “20,” consisted of 30 people of an average age 21 ± 4.3 years. The second, labelled “40,” consisted of 30 people of an average age 39 ± 8.4 years and the third, labelled “60,” consisted of 30 people of an average age 62 ± 9.1 years. All subjects were healthy nonsmokers. As a biological matrix, we have chosen exhaled breath condensate (EBC). The EBC sampling is noninvasive and can be used as a tool for diagnosis of lung diseases [97, 102]. The most significant difference in biomarker concentration can be observed for 8-iso PGF_2*α*_, but all biomarkers show a trend of an increasing concentration with increasing age. The levels of oxidative stress biomarkers in first group were 8-iso PGF_2*α*_ (15.0 ± 1.9 pg/mL EBC);* o*-Tyr (33.3 ± 3.4 pg/mL EBC); 3-ClTyr (14.5 ± 1.9 pg/mL EBC); 3-NOTyr (25.4 ± 4.8 pg/mL EBC); 8-OHdG (11.4 ± 2.1 pg/mL EBC); and 8-OHG (10.4 ± 2.7 pg/mL EBC). The second group exhibited increased values of monitored biomarkers: 8-iso PGF_2*α*_ (26.8 ± 1.9 pg/mL EBC);* o*-Tyr (41.4 ± 4.1 pg/mL EBC); 3-ClTyr (17.1 ± 2.4 pg/mL EBC); 3-NOTyr (31.3 ± 4.6 pg/mL EBC); 8-OHdG (14.9 ± 2.2 pg/mL EBC); and 8-OHG (15.7 ± 3.2 pg/mL EBC). The highest levels were confirmed in the third group: 8-iso PGF_2*α*_ (44.5 ± 5.3 pg/mL EBC);* o*-Tyr (55.6 ± 4.7 pg/mL EBC); 3-ClTyr (27.1 ± 3.2 pg/mL EBC); 3-NOTyr (43.4 ± 3.0 pg/mL EBC); 8-OHdG (24.6 ± 2.4 pg/mL EBC); and 8-OHG (32.4 ± 4.1 pg/mL EBC) (Syslova et al., unpublished results).

The study was carried out according to the Helsinki Declaration. The Ethics Committee of the 1st Faculty of Medicine, Charles University, approved all examinations and tests, and all of the study subjects gave their written informed consent for all tests and examinations.

## 5. Conclusion

Oxidative stress plays an important role in many pathological processes including age-related diseases such as atherosclerosis, hypertension, and type II diabetes. The level of oxidative damage can be measured through specific molecules, which are formed in the organism via oxidative stress. Subsequently, these substances, biomarkers of oxidative stress, not only can be monitored in body fluids and tissues of patients but also are present in healthy people in a physiological concentration. Regarding low concentrations of biomarkers in body fluids, it is necessary to choose a sensitive analytical method for the detection. A combination of separation by HPLC and detection by MS enables determination of picogram concentrations of analytes in complex biological matrices. By comparing three groups of healthy people with a different age, we found that the concentration of oxidative stress biomarkers (8-isoprostane,* o*-tyrosine, 3-chlorotyrosine, 3-nitrotyrosine, 8-hydroxy-2′-deoxyguanosine, and 8-hydroxyguanosine) is increasing with the increasing age of people. This study confirms the hypothesis that the physiological level of biomarkers depends on the age of people.

## Figures and Tables

**Table 1:** Age-related diseases and detected biomarkers.

Disease	Biomarkers	Body fluid/tissue
Alzheimer's disease	3-Chlorotyrosine	Hippocampus proteins [14]
	3-Nitrotyrosine	Brain [15]
		Cerebrospinal fluid [16, 17]
	8-Hydroxy-2′-deoxyguanosine	Brain [18]
	8-Hydroxyguanosine	Brain [18]
		Blood serum [19]
		Cerebrospinal fluid [19]

Arthritis	8-Isoprostane	Blood plasma [20]
		Urine [20]
	3-Nitrotyrosine	Blood serum [21, 22]
		Synovial fluid [22]
	8-Hydroxy-2′-deoxyguanosine	Blood plasma [23]
		Synovial fluid [23]
		Urine [24]

Atherosclerosis	3-Chlorotyrosine	Human aortic tissue [25]
	3-Nitrotyrosine	Atherosclerotic blood vessels [26]
	8-Hydroxy-2′-deoxyguanosine	Urine [27]

Cataracts	8-Isoprostane	Blood plasma [28]
	*o*-Tyrosine	Cataractous lenses [29]
	*m*-Tyrosine	Cataractous lenses [29]
	8-Hydroxy-2′-deoxyguanosine	Blood plasma [30]

Hypertension	8-Isoprostane	Blood plasma [31, 32]
	3-Nitrotyrosine	Lung tissue [33]
	8-Hydroxyguanosine	Lung tissue [33]
	8-Hydroxy-2′-deoxyguanosine	Urine [34]

Osteoporosis	8-Hydroxy-2′-deoxyguanosine	Blood serum [35]

Type II diabetes	8-Isoprostane	Blood plasma [36, 37]
		Urine [38]
	*o*-Tyrosine	Blood plasma [39]
		Urine [39]
	8-Hydroxy-2′-deoxyguanosine	Blood serum [40]
		Urine [38]

**Table 2:** Potential pathogenic role of 8-isoprostane in several diseases (reviewed in [46]).

Disease	Potential pathogenic role of 8-isoprostane
Atherosclerosis	(i) Vasoconstriction in blood vessels (ii) Influencing the aggregation of platelets (iii) Inducement of proliferation of smooth muscle cells (iv) Stimulation of proliferation of calcification blood vessel cells (v) Inhibition of differentiation of preosteoblasts

Diabetes mellitus	(i) Increase in DNA synthesis in smooth muscle cells (ii) Inducement of proliferation of smooth muscle cells (iii) Influencing membrane fluidity and permeability (iv) Renal vasoconstriction can cause systemic hypertension

Hepatorenal syndrome	(i) Renal vasoconstriction (ii) Increasing the release of endothelin-1

Preeclampsia	Renal vasoconstriction

Lung diseases	(i) Bronchoconstriction (ii) Vasoconstriction of lung artery

**Table 3:** Functional changes of nitrated proteins, adapted from [71] (shortened version).

Protein	Normal activity	Activity after nitration
Cytochrome *c *	Electron transfer and apoptosis	Higher peroxidatic activity [74, 75] Decreased apoptosome activation [78]
Fibrinogen	Coagulation	Higher aggregation [76]
Protein kinase C*ε*	Serine/threonine kinase	Translocation and activation [77]
*α*-Synuclein	Presynaptic protein	Higher aggregation [79]
Nerve growth factor	Neurotrophic factor	Neuronal apoptosis [80]
MnSOD	Superoxide dismutation	Decreased activity [72]
Prostacyclin synthase	Synthesis of prostacyclin	Decreased activity [73]
Tyrosine hydroxylase	Synthesis of L-DOPA	Decreased activity [81]
Protein kinase C	Serine/threonine kinase	Decreased activity [82]

**Table 4:** Analytical methods used for determination of age-related diseases.

Detected biomarker	Analytical method
8-Isoprostane	EIA [28, 32, 37, 38], ELISA [20, 31, 36], RIA [92–94], GC/MS [95], HPLC-MS [96], and LC-ESI-MS/MS [97]

*o*-Tyrosine	GC-MS [98–100], GC-ECD [101], HPLC-UV [29, 39], HPLC-APCI/MS/MS [101], and HPLC-MS/MS [102]

*m*-Tyrosine	GC-MS [99, 100], GC-ECD [101], HPLC-UV [39], and HPLC-APCI/MS/MS [101]

3-Chlorotyrosine	GC-MS [14, 77], GC-ECD [101], HPLC-APCI/MS/MS [101], and HPLC-ECD [103]

3-Nitrotyrosine	Immune histochemistry [33], ELISA [21], HPLC-ECD [15, 16], LC-MS/MS [17], HPLC-UV [22, 26], and HPLC-MS [22]

8-Hydroxy-2′-deoxyguanosine	Immunostaining [18], ELISA [23, 24, 34, 35, 38, 40], LC-MS/MS [27], HPLC-UV [27], and HPLC-ECD [27]

8-Hydroxyguanosine	Immunostaining [18], immune histochemistry [33], and HPLC-ECD [19]

**Table 5:** SRM transitions for the quantification of biomarkers.

Biomarker	Molecular ion [Da]	Product ion [Da]	Collision energy [eV]
8-*iso* PGF_2*α*_	352.9	193.2	27
*o*-Tyr	180.1	119.1	20
3-ClTyr	214.2	153.1	17
3-NOTyr	225.2	164.1	15
8-OHdG	282.2	192.1	21
8-OHG	298.2	208.1	20

**Table 6:** HPLC elution program. Solvent A: water solution of ammonium hydroxide (pH 10.5); solvent B: solution of methanol : acetonitrile (60 : 40, v/v) with 0.1% of ammonium hydroxide.

Time [min]	Solvent A [%]	Solvent B [%]
0:00	70	30
10:00	70	30
25:00	5	95
30:00	5	95
32:00	70	30
40:00	70	30
